# Differential Effects of Toluene and Ethanol on Dopaminergic Neurons of the Ventral Tegmental Area

**DOI:** 10.3389/fnins.2016.00434

**Published:** 2016-09-22

**Authors:** Sudarat Nimitvilai, Chang You, Devinder S. Arora, Maureen A. McElvain, Bertha J. Vandegrift, Mark S. Brodie, John J. Woodward

**Affiliations:** ^1^Department of Neuroscience, Medical University of South CarolinaCharleston, SC, USA; ^2^Department of Physiology and Biophysics, University of Illinois at ChicagoChicago, IL, USA; ^3^School of Pharmacy, Griffith UniversitySouthport, QLD, Australia

**Keywords:** reward, dopamine, electrophysiology, inhalant, volatile anesthetic

## Abstract

Drugs of abuse increase the activity of dopaminergic neurons of the ventral tegmental area (VTA), and output from the VTA is critical for both natural and drug-induced reward and reinforcement. Ethanol and the abused inhalant toluene both enhance VTA neuronal firing, but the mechanisms of this effect is not fully known. In this study, we used extracellular recordings to compare the actions of toluene and ethanol on DA VTA neurons. Both ethanol and toluene increased the firing rate of DA neurons, although toluene was ~100 times more potent than ethanol. The mixed ion channel blocker quinine (100 μM) blocked the increases in firing produced by ethanol and toluene, indicating some similarity in mechanisms of excitation. A mixture of antagonists of GABA and cholinergic receptors did not prevent toluene-induced or ethanol-induced excitation, and toluene-induced excitation was not altered by co-administration of ethanol, suggesting independent mechanisms of excitation for ethanol and toluene. Concurrent blockade of NMDA, AMPA, and metabotropic glutamate receptors enhanced the excitatory effect of toluene while having no significant effect on ethanol excitation. Nicotine increased firing of DA VTA neurons, and this was blocked by the nicotinic antagonist mecamylamine (1 μM). Mecamylamine did not alter ethanol or toluene excitation of firing but the muscarinic antagonist atropine (5 μM) or a combination of GABA antagonists (bicuculline and CGP35348, 10 μM each) reduced toluene-induced excitation without affecting ethanol excitation. The Ih current blocker ZD7288 abolished the excitatory effect of toluene but unlike the block of ethanol excitation, the effect of ZD7288 was not reversed by the GIRK channel blocker barium, but was reversed by GABA antagonists. These results demonstrate that the excitatory effects of ethanol and toluene have some similarity, such as block by quinine and ZD7288, but also indicate that there are important differences between these two drugs in their modulation by glutamatergic, cholinergic, and GABAergic receptors. These findings provide important information regarding the actions of abused inhalants on central reward pathways, and suggest that regulation of the activation of central dopamine pathways by ethanol and toluene partially overlap.

## Introduction

Dopaminergic neurons of the ventral tegmental area (DA VTA) play important roles in mediation of the rewarding and reinforcing effect of drugs of abuse (Wise, 1996; Koob and Volkow, 2010). All known drugs of abuse increase the firing rate of these neurons, and activation of the VTA and the subsequent release of dopamine in the terminal regions of the nucleus accumbens (NAc; Di Chiara and Imperato, 1988; Di Chiara et al., 2004) are well-correlated with the reinforcing actions of drugs of abuse. While recent studies have revealed the heterogeneity of the VTA and the targets of DA VTA neurons and have resulted in revisions of our understanding of the role of the VTA in addiction, it is still clear that the rewarding and reinforcing effects of all drugs of abuse are at least partially related to the release of dopamine by DA VTA neurons (Lammel et al., 2014).

Numerous studies, including those from our laboratory, have demonstrated that ethanol increases spontaneous firing of DA VTA neurons both *in vivo* (Gessa et al., 1985) and *in vitro* (Brodie et al., 1990; Brodie and Appel, 1998a,b; Xiao and Ye, 2008; Xiao et al., 2009). Ethanol directly excites DA VTA neurons, as this effect is observed in the absence of synaptic terminals (Brodie et al., 1999a) or blockers of synaptic transmission (Brodie et al., 1990). Ethanol-induced excitation of DA VTA neurons is blocked by the alkaloid quinidine (Appel et al., 2003) that shows some selectivity against delayed rectifier potassium channels, and by phorbol esters that activate certain isoforms of protein kinase C (Nimitvilai et al., 2013). Blocking h-current in DA VTA neurons antagonizes ethanol excitation (Okamoto et al., 2006), and this effect depends on activation of barium-sensitive potassium channels (McDaid et al., 2008).

Like ethanol, toluene also increases the firing rate of DA VTA neurons (Riegel and French, 1999) resulting in increases in dopamine in the nucleus accumbens (Riegel et al., 2007). Behaviorally, both toluene and ethanol act as central nervous system depressants, although at low concentrations they can produce hyperactivity. Both ethanol (Roberto et al., 2006) and toluene (Beckstead et al., 2000) have been shown to enhance GABAergic transmission either by increasing GABA release (MacIver, 2009) or by enhancing GABA_A_ receptor function (Mihic, 1999; Beckstead et al., 2000). Chronic exposure to toluene has been shown to reduce expression of the GABA_A_ alpha1 subunit expression in the VTA (Williams et al., 2005), and repeated exposures to ethanol also induce changes in GABA receptor expression (Arora et al., 2013). In addition to GABA, both toluene and ethanol potentiate serotonin 5HT_3_ function (Lovinger et al., 2000; Sung et al., 2000; Lopreato et al., 2003) and inhibit the activity of NMDA receptors (Cruz et al., 2000; Stobbs et al., 2004). Toluene also inhibits certain subtypes of the nicotinic acetylcholine receptor (Bale et al., 2002), while the ethanol induced increase of NAc dopamine *in vivo* appears to involve nicotinic cholinergic receptors located in the anterior but not posterior VTA (Ericson et al., 2008). While changes in gene expression following chronic ethanol exposure are well-studied (Mayfield et al., 2008), less progress has been made in the examination of those gene changes associated with toluene treatment. In a study with *C. elegans*, gene expression was differentially altered following chronic exposure to ethanol or toluene (Davies et al., 2012). These agents also induced different changes in locomotion with both producing decreased speed, but different patterns of movement (Davies et al., 2012). In addition, knock out of slo-1 and rab-3 eliminated the locomotor effects of ethanol, but had no effects on those produced by toluene (Davies et al., 2012).

Together, these findings suggest that there are likely some similarities and some differences in the mechanisms that underlie the increase in firing of DA VTA neurons by ethanol and toluene. As this has not been extensively studied, we compared the actions of ethanol and toluene on DA VTA neuron firing under several conditions in order to address this gap in the literature.

## Materials and methods

### Animals

Male Fischer 344 (F344; 90–150 gm) used in these studies were obtained from Harlan Sprague-Dawley (Indianapolis, IN). All rats were treated in strict accordance with the NIH Guide for the Care and Use of Laboratory Animals and all experimental methods were approved by the Animal Care Committee of the University of Illinois at Chicago.

### Preparation of brain slices

The technique for preparing brain slices containing the ventral tegmental area (VTA) has been described previously (Brodie et al., 1999b). Following rapid removal of the brain, the tissue was blocked coronally to contain the VTA and substantia nigra. The tissue block was mounted in the vibratome and submerged in chilled cutting solution. Coronal sections (400 μm thick) were cut, and the slices were placed onto a mesh platform in the recording chamber in which artificial cerebrospinal fluid (aCSF) was flowing at 2 ml/min at 35°C. The composition of the aCSF in these experiments was (in mM): NaCl 126, KCl 2.5, NaH_2_PO_4_ 1.24, CaCl_2_ 2.4, MgSO_4_ 1.3, NaHCO_3_ 26, glucose 11. The composition of the cutting solution in the vibratome was (in mM): KCl 2.5, CaCl_2_ 2.4, MgSO_4_ 1.3, NaHCO_3_ 26, glucose 11, and sucrose 220. Both solutions were saturated with 95% O_2_/ 5% CO_2_ (pH = 7.4). Equilibration time of at least 1 h was allowed after placement of tissue in the recording chamber before electrodes were placed in the tissue.

### Cell identification

The VTA was clearly visible in the fresh tissue as a gray area medial to the darker substantia nigra, and separated from the nigra by white matter. Recording electrodes were placed in the VTA under visual control. DA neurons have been shown to have distinctive electrophysiological characteristics (Grace and Bunney, 1984; Lacey et al., 1989). Only those neurons which were anatomically located within the VTA and which conformed to the criteria for DA neurons established in the literature and in this laboratory (Lacey et al., 1989; Mueller and Brodie, 1989) were studied. These criteria include broad action potentials (2.5 ms or greater, measured as the width of the bi- or tri-phasic waveform at the baseline), slow spontaneous firing rate (0.5–5 Hz), and a regular interspike interval. Cells were not tested with opiate agonists as has been done by other groups to further characterize and categorize VTA neurons (Margolis et al., 2006). It should be noted that some neurons with the characteristics we used to identify DA VTA neurons may not, in fact, be DA-containing (Margolis et al., 2006). The fact that our recordings were generally from lateral and posterior portions of the VTA and reports that most neurons in this area do in fact contain dopamine (Chieng et al., 2011), provides some confidence that neurons with the electrophysiological characteristics noted above are, in fact, dopamine-containing.

### Drug administration

When drugs were added to the aCSF, this was done by means of a calibrated infusion pump from stock solutions 100–1000 times the desired final concentrations. The addition of drug solutions to the aCSF was performed in such a way as to permit the drug solution to mix completely with aCSF before this mixture reached the recording chamber. Final concentrations were calculated from aCSF flow rate, pump infusion rate, and concentration of drug stock solution. The small volume chamber (about 300 μl) used in these studies permitted the rapid application and washout of drug solutions. Typically drugs reach equilibrium in the tissue after 2–3 min of application. Because of concerns about limited solubility and mixing of toluene into the aCSF, in some experiments, toluene was added to a separate reservoir of identical aCSF and switching between normal and toluene-containing media were achieved using a three-way tap. In other experiments, toluene was dissolved in DMSO (0.215 ml toluene in 10 ml DMSO) and added to the superfusate in the same manner as other agents. No difference in toluene response was observed when both methods were compared in the same recording (data not shown), so data using each method were pooled.

Atropine, mecamylamine, bicuculline, CGP35348, DL-AP5, CNQX, and ZD7288 were purchased from Tocris Biosciences (Minneapolis, MN). Barium chloride was purchased from Fisher Scientific (Fair Lawn, NJ). Toluene, ethanol, quinine, nicotine, and most of the salts used to prepare the extracellular media were purchased from Sigma (St. Louis, MO).

### Extracellular recording

Extracellular recording was chosen for these studies as this method permits the recordings to be stable and of long duration and allows us to assess the effects of extended exposure (>2 h) to drugs. The limitation of only measuring spontaneous action potential frequency (rather than membrane potential or other electrophysiological parameters) is counterbalanced by the advantage of being able to determine the time course of drug actions and interactions. Extracellular recording electrodes were made from 1.5 mm diameter glass tubing with filament and were filled with 0.9% NaCl with the addition of drugs of interest or appropriate control vehicle. Tip resistance of the microelectrodes ranged from 2 to 4 MΩ. A high-gain extracellular amplifier was used in conjunction with an IBM-PC-based data acquisition system (ADInstruments, Inc.). Offline analysis was used to calculate, display and store the frequency of firing 1 min intervals. Additional software was used to calculate the firing rate over 5 s intervals. Firing rate was determined before and during drug application. Firing rate was calculated over 1 min intervals prior to administration of drugs and during the drug effect; peak drug-induced changes in firing rate were expressed as the percentage change from the control firing rate according to the formula [(FRD — FRC)/FRC] × 100, where FRD is the firing rate during the peak drug effect and FRC is the control firing rate. The change in firing rate thus is expressed as a percentage of the initial firing rate, which controls for small changes in firing rate which may occur over time. This formula was used to calculate both excitatory and inhibitory drug effects. Peak excitation was defined as the peak increase in firing rate produced by the drug (e.g., toluene) greater than the pre-drug baseline. Inhibition was defined as the lowest firing rate below the pre-drug baseline.

### Data collection

For comparison of the time course of effects on firing rate, the data were normalized and averaged. Firing rates over 1 min intervals were calculated and normalized to the 1 min interval immediately prior to the DA administration. These normalized data were averaged by synchronizing the data to the DA administration period, and graphs of the averaged data were made.

### Statistical analysis

Averaged numerical values were expressed as the mean ± the standard error of the mean (S.E.M.). The differences among firing rates during the long drug administration intervals in these studies was assessed using *t*-tests, or with an appropriate ANOVA followed by Tukey *post-hoc* comparisons (Kenakin, 1987). Statistical analyses were performed with GraphPad Prism version 6.05 (GraphPad Software, Inc., La Jolla, CA).

## Results

A total of 123 VTA neurons were recorded in this study. Their initial firing rate ranged from 0.67 to 4.27 Hz, with a mean of 1.93 ± 0.06 Hz. All neurons had regular firing rates, and conformed to the rate and patterns of DA VTA neurons as described in the Methods above.

### Toluene concentration-response

**Figure 1:**
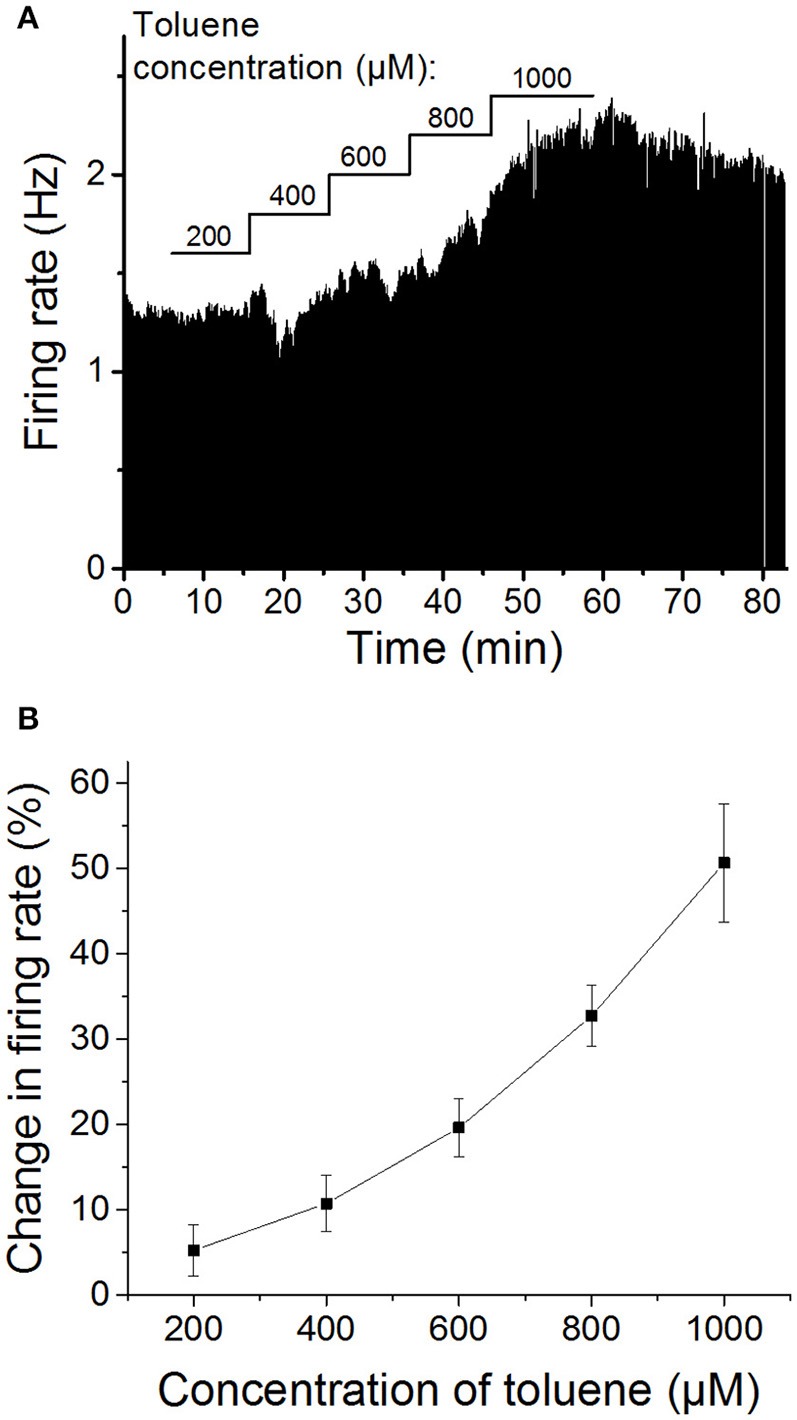
**Concentration-dependent excitation of DA VTA neurons by toluene**. **(A)** Ratemeter graph of firing rate over time recorded from a single VTA neuron. Vertical bars indicate firing rate over a 5 s interval; horizontal bars indicate the duration of administration of toluene (concentration indicated above bar). Toluene produced increases in firing (in Hz) of 200 μM: 1.01; 400 μM: 7.35; 600 μM: 15.87; 800 μM: 38.16; 1000 μM: 66.33. **(B)** Pooled concentration-response curve of the effect of toluene on spontaneous activity of DA VTA neurons. Toluene was added to the superfusate and concentrations were increased in a step-wise manner (10 min per step) from 200 to 1000 μM in 200 μM steps. (These same data are shown in Figure 4A below as control).

As has been shown by others (Riegel and French, 2002), we initially confirmed that toluene increases firing of VTA DA neurons in a dose-dependent manner. Following a stable baseline period, five concentrations of toluene were tested, beginning with 200 μM toluene and increasing the concentration in a stepwise fashion (200, 400, 600, 800, and 1000 μM), with each concentration being applied for 10 min. As shown in Figure 1, toluene induced a concentration-dependent increase in firing rate that was significantly different from baseline [one-way repeated measures ANOVA, *F*_(1.6, 11.3)_ = 19.77, *P* < 0.0005, *n* = 8]. Note that in some experiments, an initial inhibition was observed prior to the excitatory response. We have observed this with ethanol as well (Brodie et al., 1990), and these inihibitory responses do not appear to be concentration-dependent (see Figure 1A) and may be induced by actions of toluene or ethanol on neurons in the vicinity of the cell that is the subject of the recording. Despite the clear concentration-dependence of toluene-induced excitation, the individual responses were quite variable; for example, the range of responses to 600 μM toluene was 8.7–35.2% excitation, and for 1000 μM toluene, the range was 21.1–80.6% excitation. As noted above, recordings were made from lateral VTA neurons in the posterior portion of the VTA; we did not observe any differences in the response to ethanol or toluene dependent on the location of the neurons in the slice preparation.

### Quinine blocks the excitatory effects of ethanol and toluene

**Figure 2:**
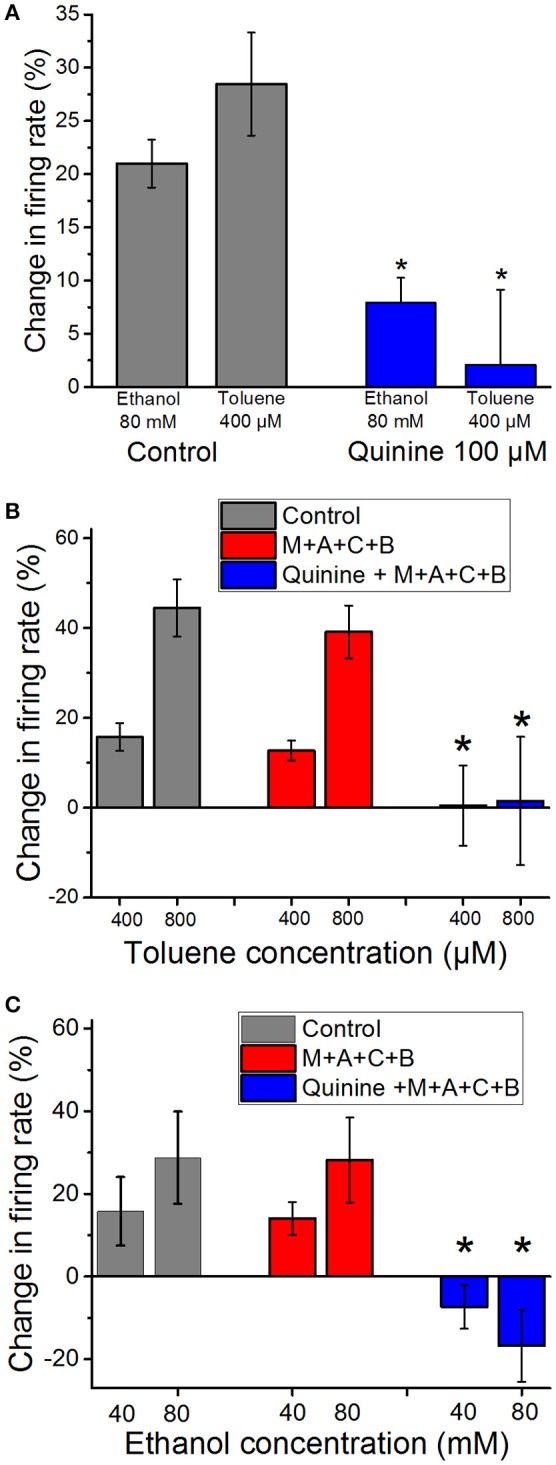
**Reduction of ethanol- and toluene-induced excitation by quinine (A)**. Mean responses to ethanol (80 mM) and toluene (400 μM) in the absence (gray bars) and presence of 100 μM quinine (red bars). Quinine significantly reduced the excitation produced by toluene and ethanol, suggesting some overlap in the mechanisms of excitation by both drugs (^*^paired *t*-test, *P* < 0.01). **(B)** Toluene (400 or 800 μM) was tested before (gray bars) and in the presence of a cocktail (**M**+**A**+**C**+**B**) of mecamylamine (1 μM), atropine (5 μM), bicuculline chloride (10 μM), and CGP35348 (10 μM; red bars). The effect of toluene was not altered significantly by the addition of these antagonists (*n* = 9). In four of these experiments, after toluene was tested in the antagonists, 100 μM quinine (blue bars) was added to the superfusate and toluene was retested (**Quinine** + **M**+**A**+**C**+**B**). Toluene-induced excitation was significantly reduced in the presence of quinine and the four antagonists [^*^two-way repeated measures ANOVA, *F*_(2, 6)_ = 14.76, *P* < 0.005, *n* = 4]. **(C)** Ethanol (40 or 80 mM) was tested before (gray bars) and in the presence of a cocktail of mecamylamine (1 μM), atropine (5 μM), bicuculline chloride (10 μM), and CGP35348 (10 μM; red bars). The effect of ethanol was not altered significantly by the addition of these antagonists (*n* = 5). In addition, after ethanol was tested in the antagonists, 100 μM quinine (blue bars) was added to the superfusate and ethanol was retested. Ethanol-induced excitation was significantly reduced in the presence of quinine and the four antagonists [^*^two-way RM ANOVA, *F*_(2, 8)_ = 15.27, *P* < 0.002: Tukey *post-hoc* comparison of means, *P* < 0.05 for significance; *n* = 5].

We have shown previously that ethanol-induced excitation of DA VTA neuron firing is antagonized by the alkaloid quinidine, that, like its stereoisomer quinine, has some selectivity for a variety of delayed rectifier K^+^ channels (Snyders et al., 1992; Yeola et al., 1996; Singh and Singh, 1999; Schönherr et al., 2002; Appel et al., 2003), although it can block sodium (Grant et al., 1984) and calcium (Salata and Wasserstrom, 1988) channels as well. In the present study we tested whether quinine could antagonize ethanol- and toluene-induced excitation. As shown in Figure 2A, ethanol (80 mM) and toluene (400 μM) each produced 20–30% increase in firing of DA VTA neurons. Quinine (100 μM) significantly reduced excitation induced by both ethanol (paired *t*-test, *P* < 0.01) and toluene (paired *t*-test, *P* < 0.01). This finding suggests that there is some overlap in the mechanisms of excitation induced by ethanol and toluene in that they might both excite DA VTA neurons through blocking quinine-sensitive K^+^ currents.

### Concurrent blockade of cholinergic and GABAergic receptors

Evidence in the literature suggests that toluene can interact with both cholinergic and GABAergic systems. In order to test whether either of these systems play a direct role in toluene-induced excitation of DA VTA neurons, we tested and compared the effects of toluene and ethanol in the absence or presence of antagonists of GABA (10 μM bicuculline; 10 μM CGP35348) and cholinergic (1 μM mecamylamine and 5 μM atropine) receptors. The combination of antagonists alone produced a significant increase in baseline firing rate of 17.3 ± 4.5% (paired *t*-test, *t* = −3.088, DF = 8, *P* < 0.02; *n* = 9; Table 1). In the toluene experiments, two concentrations (400 and 800 μM) of toluene were tested, then the combination of antagonists was added to the superfusate, and the same two concentrations of toluene were retested (*n* = 9). In a subset of experiments (*n* = 4), after testing toluene in the presence of the GABA and cholinergic antagonists, quinine (100 μM) was added to the superfusate, and the same concentrations of toluene were tested again in the presence of the four receptor antagonists and quinine. As shown in Figure 2B, there was no significant change in the excitatory action of either dose of toluene on DA VTA neurons in the mixture of GABA and cholinergic antagonists. However, the toluene-induced increase in firing in the presence of the antagonists was blocked by quinine [two-way ANOVA, *F*_(2, 6)_ = 14.762, *P* < 0.005: Tukey *post-hoc* comparison of means, *P* < 0.05 for significance]. In a separate group of neurons, we also tested the effect of the antagonists and quinine on ethanol-induced excitation. Similar to the results with toluene, the antagonist mixture had no significant effect on the excitatory action of either 40 or 80 mM ethanol (Figure 2C). Under these conditions, ethanol-induced excitation was also blunted when quinine was added [two-way repeated measures ANOVA, *F*_(2, 8)_ = 15.27, *P* < 0.002: Tukey *post-hoc* comparison of means, *P* < 0.05 for significance]. These results confirmed that excitation by both ethanol and toluene are mediated by a quinine-sensitive process and suggest that the excitation of DA VTA neurons by these drugs cannot be mediated directly by either GABA or cholinergic neurotransmission.

**Table 1:** **Effects of various antagonists on firing rate**.

**Figure number: antagonist (s)**	**Change in firing rate (% ± SEM)**	**Significance**
2A: quinine	−17.6% ± 12.0	*P* > 0.05
2B: MACB	17.3 ± 4.5	*P* < 0.02
2C: MACB	6.2 ± 4.0	*P* > 0.05
3: MACB	2.0 ± 3.4	*P* > 0.05
3: MACB + ethanol	19.6 ± 2.6	*P* < 0.0001
4A: glutamate antagonists	−2.97 ± 3.8	*P* > 0.05
4B: glutamate antagonists	7.43 ± 3.05	*P* < 0.05
5B: mecamylamine	−0.09 ± 6.16	*P* > 0.05
5C: atropine	21.0 ± 6.7	*P* > 0.05
5D: GABA antagonists	9.89 ± 3.08	*P* < 0.01
6: barium	58.02 ± 16.3	*P* < 0.005

*MACB, mecamylamine (1 μM); atropine (5 μM); CGP35348 (10 μM); bicuculline (10 μM)*.

*Glutamate antagonists, DL-AP5 (10 μM); CNQX (10 μM); MCPG (10 μM)*.

*The effect of antagonists used in these experiments on firing rate, referenced to figure number. In some cases, different groups of cells responded differently with the same combination of antagonists due to cell-to-cell variability*.

### Toluene excitation in the presence of ethanol

**Figure 3:**
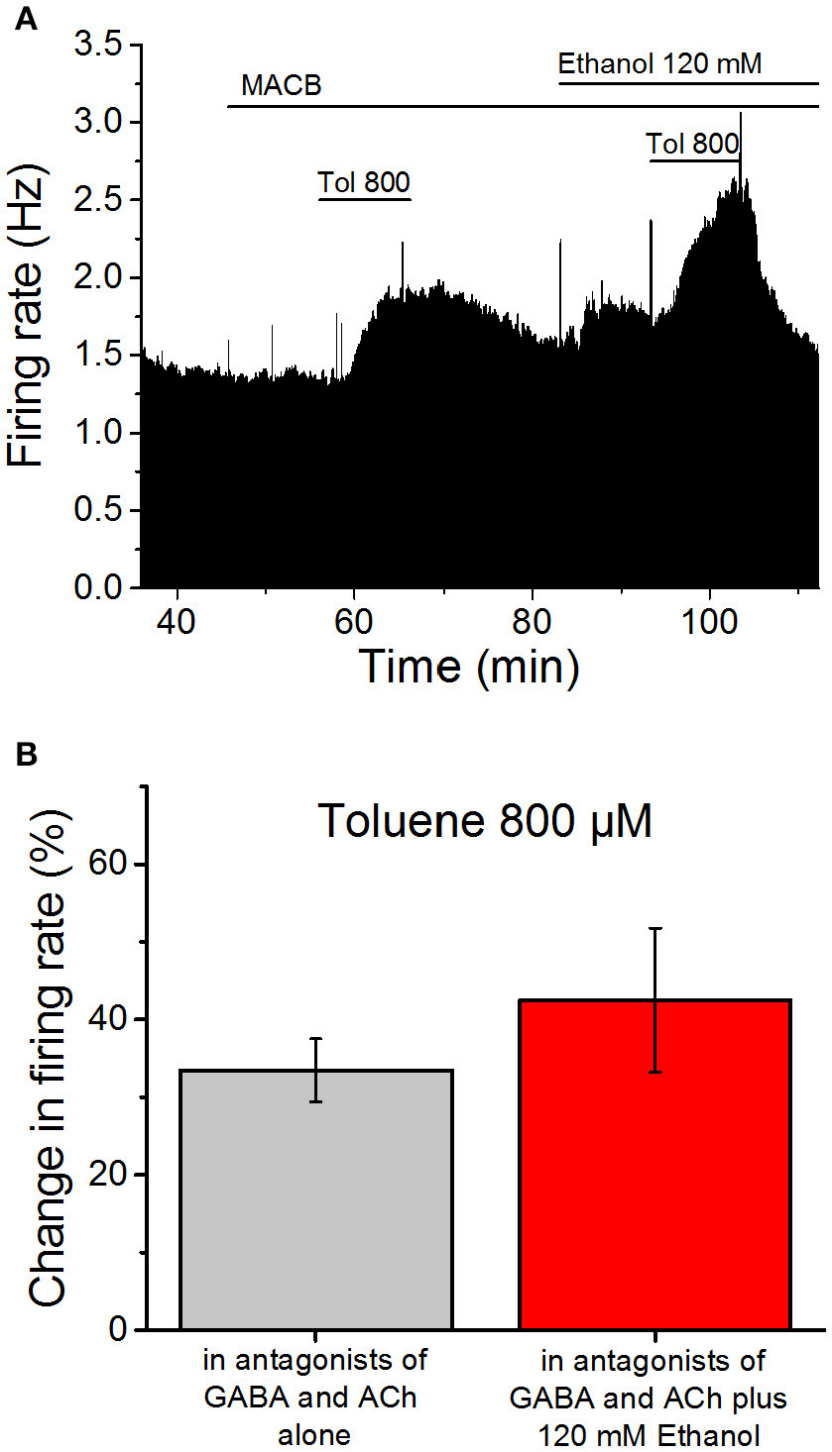
**Ethanol does not occlude toluene-induced excitation. (A)** Ratemeter diagram illustrating the results from a single VTA neuron undergoing the protocol used in the ethanol occlusion experiments. Vertical bars indicate the firing rate over 5 s intervals, and horizontal bars indicate the duration of drug administration (specific drugs indicated above these bars). After beginning a recording, the same antagonist cocktail described in the legend to Figure 2 was added to the superfusate. Toluene (800 μM) was tested and then washed out. Then ethanol (120 mM) was added to the superfusate and, after 10 min, the same concentration of toluene was tested again. In this recording, toluene increased the firing rate by 34.0% prior to ethanol and 39.5% in the presence of ethanol, while ethanol alone produced a 15.4% increase in firing rate. **(B)** Toluene (800 μM) was tested before (gray bar) and in the presence of 120 mM ethanol (red bar). Note that a cocktail of mecamylamine (1 μM), atropine (5 μM), bicuculline chloride (10 μM) and CGP35348 (10 μM) is present during the whole experiment. The effect of toluene was not altered significantly by the presence of ethanol (*n* = 8).

If toluene and ethanol act on the same quinine-sensitive membrane protein, then applying ethanol first should decrease the subsequent excitatory effect of toluene. We therefore tested the effect of 800 μM toluene in the absence and presence of 120 mM ethanol, as these concentrations produce roughly equivalent effects on firing. Similar to the studies discussed above, these studies were performed in the presence of antagonists of cholinergic (1 μM mecamylamine; 5 μM atropine) and GABAergic (10 μM bicuculline; 10 μM CGP35348) receptors (Figure 3A). Toluene was tested, washed out, and then 120 mM ethanol was added to the superfusate. Ten minutes after the addition of ethanol, toluene was added in the presence of ethanol. The mean data shown in Figure 3B illustrate that, in the presence of the antagonists, 800 μM toluene increased firing frequency by 33.4 ± 4.1%. Following washout of toluene, addition of 120 mM ethanol increased firing by 19.6 ± 2.6% (data not shown). In the presence of ethanol, toluene increased firing by 42.5 ± 9.3%, an amount that was not significantly different from that observed in the absence of ethanol (paired *t*-test, DF = 7, *t*-statistic = −0.994, *n* = 8). The lack of occlusion of toluene-induced excitation by a high ethanol concentration suggests that ethanol and toluene may act at different sites on DA VTA neurons.

### Effects of glutamate antagonists

**Figure 4:**
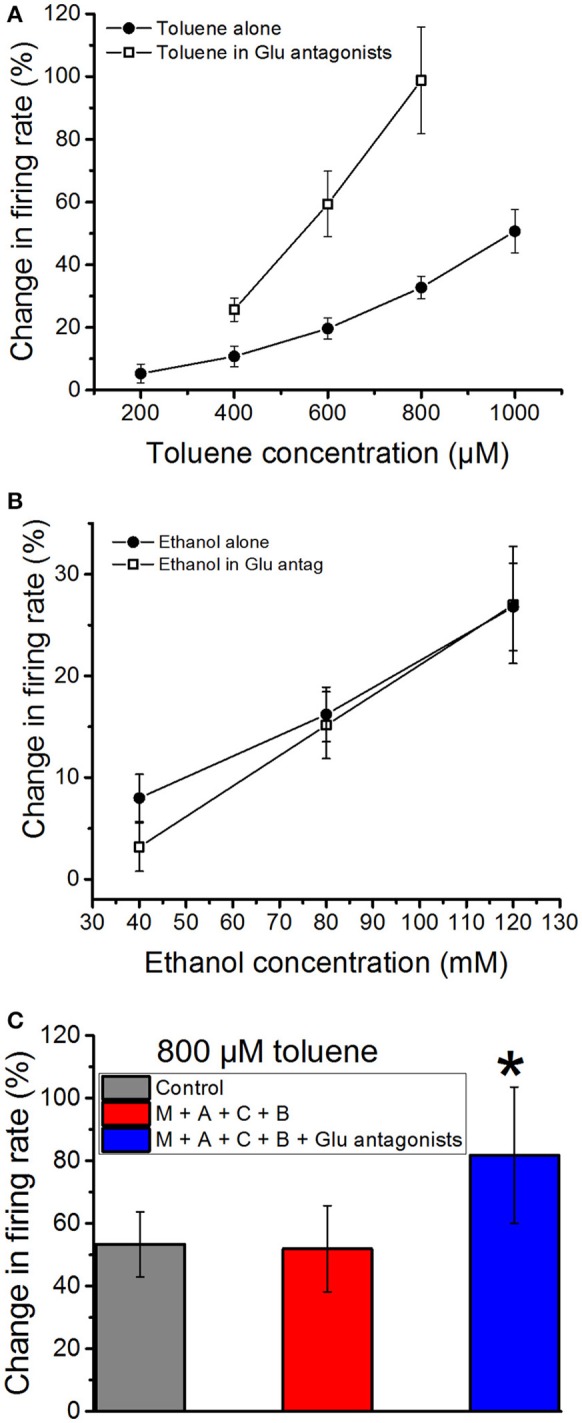
**Effects of ethanol and toluene in the presence of glutamate antagonists**. Toluene **(A)** and ethanol **(B)** were tested in the absence or presence of a combination of NMDA antagonist DL-AP5 (10 μM), AMPA receptor antagonist CNQX (10 μM), and mGluR antagonist MCPG (10 μM). **(A)** Toluene alone excited the DA VTA neurons in a concentration-dependent manner. In the presence of glutamate antagonists, there was a significant increase in the excitatory effects of 400–800 μM toluene. **(B)** Ethanol (40–120 mM) produced a concentration-dependent excitation. In the presence of glutamate antagonists, ethanol-induced excitation was not changed significantly. **(C)** Toluene excitation in the presence of antagonists of cholinergic, GABAergic, and glutamatergic receptors. The effect of 800 μM toluene alone (gray bar) and in the presence of a cocktail of mecamylamine (1 μM), atropine (5 μM), bicuculline chloride (10 μM), and CGP35348 (10 μM; red bar) and that cocktail plus a combination of NMDA antagonist DL-AP5 (10 μM), AMPA receptor antagonist CNQX (10 μM), and mGluR receptor antagonist MCPG (10 μM; blue bars). There was no difference in the response to toluene in the presence of the cholinergic and GABAergic antagonists, but there was a significant increase in the response after addition of the glutamate antagonists [^*^P < 0.05, one-way repeated measures ANOVA, *F*_(1.3, 11.3)_ = 5.50, *n* = 10].

As glutamate is the predominant excitatory neurotransmitter in the VTA *in vivo* (Kalivas, 1993), we next tested whether blockade of glutamate receptors would alter the excitatory actions of toluene or ethanol. A combination of an NMDA antagonist DL-AP5 (10 μM), an AMPA receptor antagonist CNQX (10 μM), and an mGluR antagonist MCPG (10 μM) was added to the superfusate; this cocktail produced only a small, non-significant (−2.97 ± 3.8%) change in firing rate. This suggests that, in the acute brain slice preparation, glutamate has only a modest role in maintaining the steady-state spontaneous activity of DA VTA neurons. In contrast, the excitatory effect of toluene was significantly increased in the presence of the glutamate receptor antagonists as compared to the no-antagonist condition [two-way ANOVA, *F*_(1, 41)_ = 37.01, *P* < 0.01, *n* = 8; Figure 4A]. The analysis also revealed a significant interaction between toluene concentration and antagonist [two-way repeated measures ANOVA, *F*_(2, 41)_ = 4.9, *P* < 0.02]. In contrast to these results, the effect of ethanol on firing was not significantly altered by the glutamate receptor antagonists [two-way ANOVA, *F*_(1, 51)_ = 0.354, *P* > 0.05; Figure 4B]. To determine whether the enhanced effect of toluene in the presence of the glutamate receptor antagonists was dependent upon other neurotransmitter receptors, we examined the effect of toluene in the presence of antagonists of glutamate, GABA, and acetylcholine. As shown in Figure 4C, 800 μM toluene alone produced a 53.3 ± 10.3% increase in firing. Consistent with the results shown in Figure 2B, in the presence of the cholinergic and GABAergic antagonists, 800 μM toluene produced a 51.9 ± 13.7% increase in excitation. In the presence of the GABA/ACh antagonists, adding the glutamatergic antagonists resulted in a toluene-induced increase in excitation to 81.8 ± 21.7%. There was a significant difference in the response to toluene in the presence of all of the antagonists compared to the other conditions [one-way repeated measures ANOVA, *F*_(1.3, 11.3)_ = 5.50, *P* < 0.05; *n* = 10; Tukey *post-hoc* comparison of means, *P* < 0.05 for significance], but, again, no difference in the response to toluene in the absence or presence of the combination of cholinergic and GABAergic antagonists. Therefore, toluene-induced excitation is enhanced in the presence of antagonists of the glutamate receptor.

### Antagonists of acetylcholine and GABA reduce excitation produced by toluene but not that produced by ethanol

**Figure 5:**
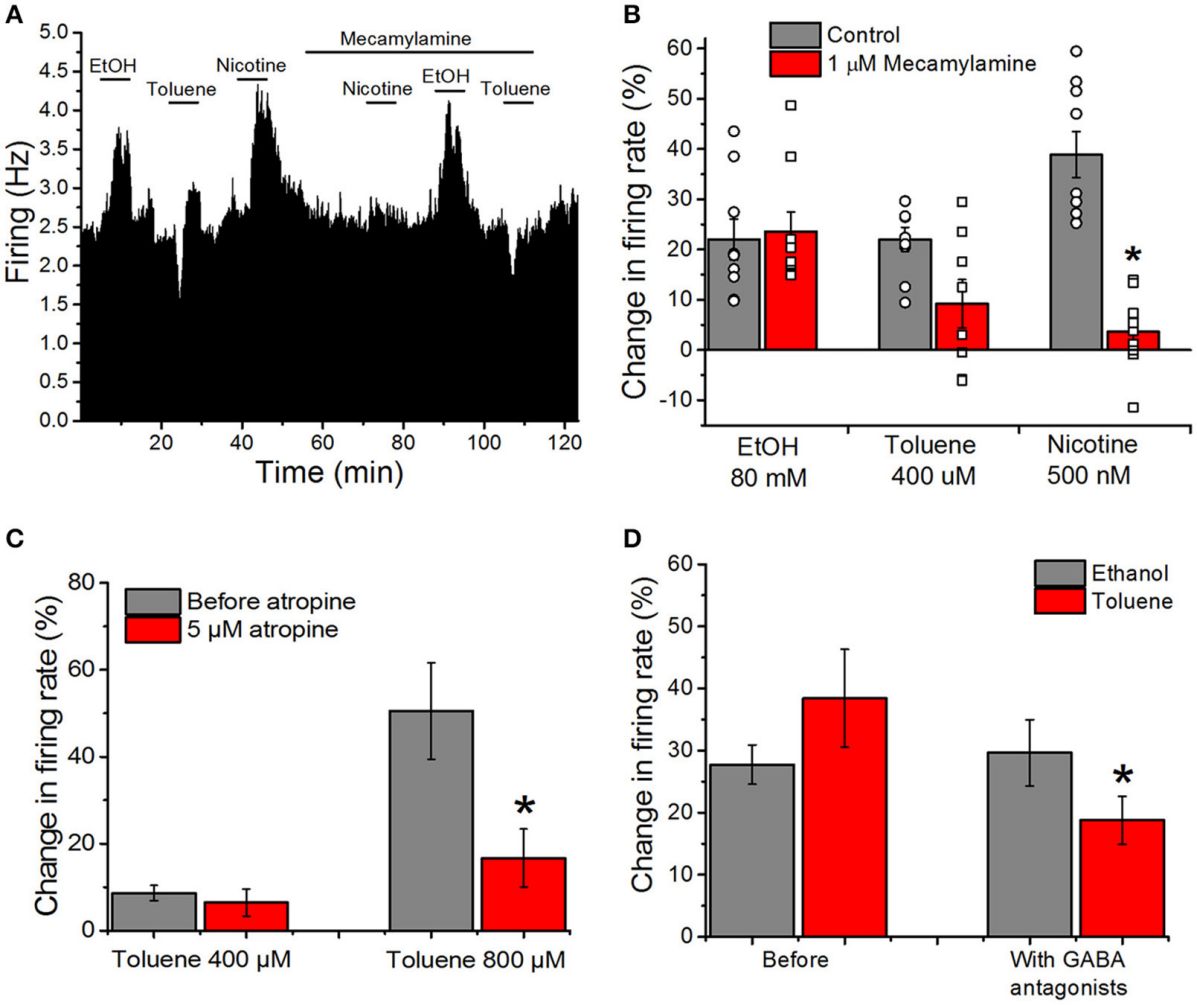
**Cholinergic and GABAergic antagonists reduce toluene-induced excitation, but have no effect on excitation produced by ethanol. (A)** Ratemeter: Firing rate over 5 s intervals is represented by the height of the vertical bars; duration of drug application is shown by the horizontal bars. Ethanol (EtOH, 80 mM), toluene (400 μM) and nicotine (500 nM) were tested before and in the presence of mecamylamine (1 μM). All three drugs produced excitation in the absence of mecamylamine, but in the presence of mecamylamine, there was a reduction in the excitation produced by nicotine and toluene, with no diminution of the ethanol-induced excitation. Despite the reduction in toluene-induced excitation by mecamylamine in this recording, there was no significant effect of mecamylamine on toluene excitation in the population of responses studied overall. **(B)** Bars representing the mean responses to ethanol, toluene, and nicotine in the absence (gray bars) and presence (red bars) from the pool of experiments similar to the one depicted in **(A)**. Due to the variation in responses in the presence of mecamylamine, individual responses in the absence (round symbols) and presence (square symbols) are shown superimposed on the bars. Mecamylamine significantly reduced the excitation produced by nicotine but not that produced by toluene or ethanol. **(C)** Toluene (400 or 800 μM) was tested before (gray bars) and after the addition of 5 μM atropine to the superfusion medium (red bars). In the presence of atropine, the effects of 800 μM toluene were significantly reduced [two-way ANOVA, *F*_(1, 3)_ = 20.74, *P* < 0.02, *n* = 4]. **(D)** The effects of ethanol (80 mM; gray bars) and toluene (400–800 μM; red bars) were assessed before and after addition of the GABA_A_ antagonist bicuculline (10 μM) and GABA_B_ antagonist CGP35348 (10 μM) to the superfusion medium. Ethanol-induced excitation was not significantly altered by the GABA antagonists, but the excitation produced by toluene was significantly reduced [^*^P < 0.05, two-way ANOVA, *F*_(1, 13)_ = 10.44, *P* < 0.01, *n* = 14].

Despite the lack of effect of cholinergic and GABAergic antagonists in combination on toluene or ethanol excitation (Figures 2B,C), we chose to examine cholinergic and GABAergic antagonists independently as well. Our previous reports suggest that toluene inhibits both recombinant and native nicotinic cholinergic receptors (Bale et al., 2002, 2005). To test whether nAChRs on the VTA are involved in the alteration of firing rate by toluene and ethanol, we recorded responses in the absence and presence of the selective nicotinic antagonist mecamylamine. A typical experiment is shown in Figure 5A and the mean responses are shown in Figure 5B. Before mecamylamine was added, ethanol (80 mM), toluene (400 μM), and nicotine (500 nM) were tested and each increased the firing rate. Mecamylamine (1 μM) had no effect on its own, but it significantly blunted the increase caused by nicotine [two-way ANOVA, *F*_(2, 46)_ = 23.26, *P* < 0.001; *n* = 9] while having no significant effect on toluene or ethanol excitation. It should be noted that while only one of nine neurons showed reduction of ethanol excitation by mecamylamine, in 4 of 8 neurons tested, mecamylamine completely or nearly completely blocked toluene excitation. Sensitivity to mecamylamine was not dependent on initial firing rate, and all of the neurons were located in a similar region of the VTA.

As DA VTA neurons also have muscarinic cholinergic receptors (Lacey et al., 1990), we determined whether blocking muscarinic receptors with atropine would alter toluene-induced excitation. Toluene (400 and 800 μM) was tested in the absence and presence of 5 μM atropine; atropine alone increased firing rate by ~21% (Table 1). In the presence of atropine, toluene excitation of firing was significantly reduced and this was especially apparent at the higher toluene concentration [two-way ANOVA, *F*_(1, 3)_ = 20.74, *P* < 0.02, *n* = 4; Figure 5C]. This finding suggests that there may be cholinergic interactions with toluene-induced excitation.

There is a large GABAergic innervation of DA VTA neurons (Bayer and Pickel, 1991), and GABAergic neurotransmission plays an important role in regulating the firing of VTA DAergic neurons (Theile et al., 2008, 2011; Xiao and Ye, 2008; Steffensen et al., 2009). We tested whether excitation induced by either ethanol or toluene was affected by GABA antagonists. The excitatory effects of 80 mM ethanol and 400–800 μM toluene were tested before and after the addition of GABA antagonists to the external medium. Figure 5D illustrates the effect of addition of the GABA_A_ antagonist bicuculline (10 μM), and the GABA_B_ antagonist CGP35348 (10 μM) on toluene and ethanol excitation. GABA_A_ antagonist bicuculline plus CGP35348 produced a small but significant increase in firing rate (Table 1). Prior to adding the GABA antagonists, ethanol excitation was 27.7 ± 3.2% and this did not change in the presence of the GABA antagonists (29.6 ± 5.4%). In contrast, under control conditions, toluene excitation was 38.4 ± 7.9% and this declined to 18.8 ± 3.9% when the recordings were carried out in the presence of GABA antagonists [two-way ANOVA, *F*_(1, 13)_ = 10.44, *P* < 0.01, *n* = 14; Figure 5D]. These results indicate that concurrent block of GABA_A_ and GABA_B_ receptors can modulate toluene-induced excitation, and illustrates another difference between toluene and ethanol.

### Effect of toluene in the presence of ZD7288

**Figure 6:**
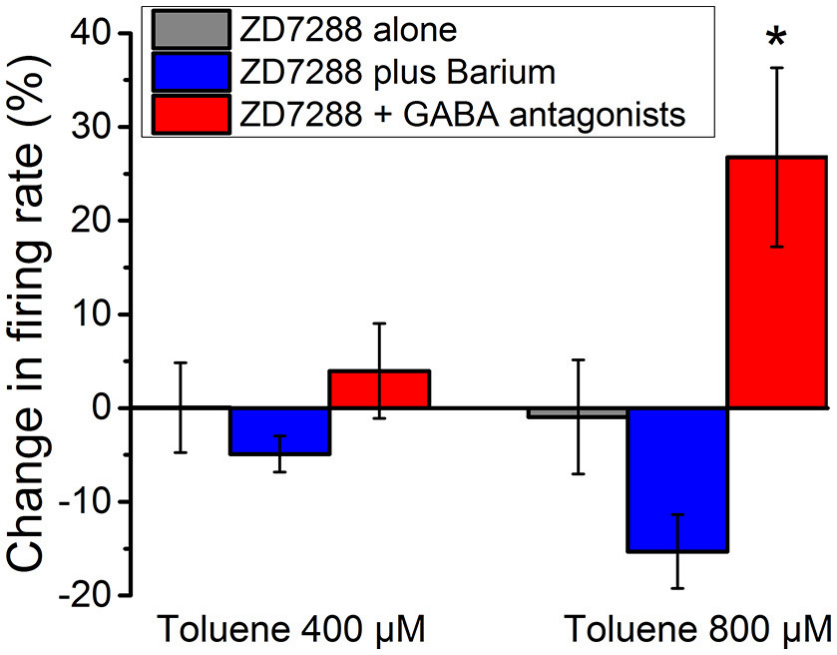
**Effect of toluene in the presence of ZD7288 and GABA antagonists**. Toluene (400 or 800 μM) was tested in the presence of ZD7288 (20 μM) (gray), ZD7288 plus 100 μM barium (blue), or ZD7288 and a combination of GABA antagonists [GABA-A antagonist bicuculline (10 μM) and GABA-B antagonist CGP35348 (10 μM); red]. With ZD7288 in the medium, there was an apparent blockade of the excitatory effects of toluene. This blockade of toluene-induced excitation was not reversed by the addition of barium to the medium. In the presence of ZD7288 and GABA antagonists, there was a significant restoration of the toluene-induced excitation (*P* < 0.001; two-way Anova, *F*_(2, 40)_ = 9.24; ^*^P < 0.05).

DA VTA neurons possess hyperpolarization-activated cyclic nucleotide-gated current (h-current) that has been demonstrated to be sensitive to ethanol (Brodie and Appel, 1998b; Okamoto et al., 2006; McDaid et al., 2008). Previous studies have demonstrated that ethanol excitation of DA VTA neurons is reduced in the presence of the selective h-channel blocker, ZD7288 (Okamoto et al., 2006; McDaid et al., 2008). This reduction of ethanol excitation results from the opening of ethanol-sensitive G protein-coupled inwardly rectifying potassium (GIRK) channels; in the presence of the GIRK channel blocker barium, ZD7288 no longer affects ethanol excitation (McDaid et al., 2008). Therefore, inhibitory barium-sensitive currents that are activated by ethanol are modulated by h-current, and blockade of h-current by ZD7288 reveals this inhibition that counteracts the excitatory effect of ethanol (McDaid et al., 2008). For comparison with these published results, we examined the action of toluene in the presence of ZD7288. The excitatory effect of 400 and 800 μM toluene was essentially eliminated by 20 μM ZD7288 (Figure 6, gray bars, *n* = 10), but, unlike our results with ethanol, the effect of ZD7288 on toluene-induced firing was not reversed by the GIRK channel blocker barium chloride (Figure 6, blue bars). However, toluene-induced excitation of firing was again observed if ZD7288 was co-administered with antagonists of GABA_A_ (bicuculline 10 μM) and GABA_B_ (CGP35348 10 μM) receptors [Figure 6, red bars; two-way ANOVA, *F*_(2, 40)_ = 9.24, *P* < 0.001; Tukey *post-hoc* comparison of means, *P* < 0.05 for significance]. These data indicate that while the h-channel blocker ZD7288 can appear to block toluene-induced excitation, similar to its effect on ethanol-induced excitation, this action requires functional GABA_A_ and GABA_B_ receptors.

## Discussion

Previous studies show that toluene (Riegel and French, 2002; Riegel et al., 2007) and ethanol (Brodie et al., 1999a) increase the firing rate of dopaminergic neurons of the VTA. In the present study, we compared the responses of DA VTA neurons to ethanol and toluene under similar experimental conditions, and in many cases the same neurons. In summary, the results of this study show that the excitation produced by either drug is blocked by quinine and that the actions of both drugs are regulated, albeit differently, by ZD7288-sensitive h-current. The lack of reduction of toluene excitation in the presence of ethanol suggests that these drugs act on different molecular targets. Muscarinic cholinergic receptors and GABA receptors appear to regulate toluene, but not ethanol, excitation. Finally, while our previously published work demonstrated that block of h-currents allows for ethanol inhibition of firing via opening of barium-sensitive potassium channels (McDaid et al., 2008), we show here that blocking h-current uncovers a toluene-induced activation of GABA-activated channels.

Results from the various pharmacological antagonist experiments performed in the present study (Table 2) revealed significant differences between ethanol and toluene in the regulation of DA VTA neuronal excitability. Interestingly, the combination of GABA_A_ and GABA_B_ antagonists had no effect on ethanol-induced excitation, but significantly suppressed excitation produced by toluene, and when applied alone, neither GABA_A_ nor GABA_B_ antagonists significantly affected toluene-induced excitation. This observation could reflect a complex interaction between excitatory and inhibitory actions of toluene on GABA neurotransmission. This complexity is underscored further by the lack of effect of a combination of GABAergic and cholinergic antagonists on toluene-induced excitation. The results indicate that neither cholinergic nor GABAergic processes play direct roles in toluene-induced excitation, but may serve to modulate that excitation.

**Table 2:** **Summary of the effects of agents tested in this study**.

	**Toluene**	**Ethanol**
Quinine	reduced	reduced
MACB combination	no effect	no effect
Ethanol	no effect	N/A
Glutamate antagonists	Increased	no effect
MACB combination + Glu antagonists	Increased	no effect
Mecamylamine	no effect	no effect
Atropine	decreased	no effect
ZD7288	decreased	decreased
ZD7288 + barium	decreased	no effect^*^
ZD7288 + GABA antagonists	no effect	not tested

*MACB, mecamylamine (1 μM); atropine (5 μM); CGP35348 (10 μM); bicuculline (10 μM)*.

*Glutamate antagonists, DL-AP5 (10 μM); CNQX (10 μM); MCPG (10 μM)*.

* *Tested previously by our laboratory in McDaid et al. (2008)*.

*Comparison of the effects of antagonists on ethanol and toluene excitation*.

One point that bears further examination is the lack of effect of the combination of receptor antagonists (Figure 2, MACB conditions), despite the significant effects of subsets of antagonists (GABA antagonists, for example) to reduce toluene-induced excitation. Likewise, the lack of effect of either GABA antagonist alone despite the significant effect of the combination is somewhat perplexing. We suggest that these observations are gemaine to studies of ethanol as well as toluene, and illustrates the difference between antagonism of a primary mechanism and antagonism of a modulatory influence. Numerous studies have suggested that ethanol excitation is mediated by cholinergic (Larsson et al., 2002), GABAergic (Ludlow et al., 2009; Theile et al., 2011; Guan et al., 2012), glutamatergic (Xiao et al., 2009), or combinations of neurotransmitter systems (Engle et al., 2015). We previously have shown that ethanol excites acutely isolated DA VTA neurons that were enzymatically treated to eliminate synaptic contacts (Brodie et al., 1999a), indicating neither neurotransmitter receptor activation nor blockade is needed to produce ethanol excitation. In concurrence with that earlier study, the present study demonstrates that none of the receptor antagonist combinations significantly reduced ethanol excitation. For both ethanol and toluene, the h-channel blocker ZD7288 produces an apparent antagonism of ethanol (Okamoto et al., 2006) and toluene (Figure 2B) excitation. In the case of ethanol, this antagonism is reversed by barium (McDaid et al., 2008) and in the case of toluene, this antagonism is reversed by GABA blockers (Figure 6). In both cases, ZD7288 is not blocking the excitatory mechanism, but is revealing an inhibitory component of the response to ethanol or toluene. The effects of these inhibitory responses are muted or silenced by the shunting of inhibitory current by h-channels. Quinine [and quinidine in the case of ethanol (Appel et al., 2003)] blocked the excitation in a more direct manner; all of the other effective antagonists blocked a modulatory influence that affected the expression of the excitation without directly altering the excitation itself. Unfortunately, quinine has actions on many ion channels, including delayed rectifier potassium channels, sodium channels, and calcium channels thus preventing absolute identification of the toluene sensitive component.

The information about neurotransmitter receptor roles in modulation of toluene excitation may be relevant to associated effects of toluene in the VTA not directly related to the excitatory action. In addition to a GABA-related excitatory effect of toluene (perhaps via disinhibition of GABAergic inputs onto the DA VTA neurons), the results from the ZD7288 experiments suggest that toluene activates GABA_A_ channels, an effect that becomes more significant when h-current is blocked. As h-current is activated by negative membrane potentials, any hyperpolarizing event (such as opening of GABA_A_ channels) would likely increase h-current, thereby shunting the effects of the inhibitory current. Blocking h-current with ZD7288 thus would prevent that shunting and reveal an inhibitory action. This hypothesis is the same as one we previously offered to explain the action of ZD7288 on ethanol excitation (McDaid et al., 2008). In the case of ethanol, barium restored ethanol-induced excitation of DA VTA neurons, suggesting an ethanol-induced opening of barium-sensitive channels, a finding consistent with previous studies showing GIRK activation by ethanol (Lewohl et al., 1999). In the case of toluene, adding barium with ZD7288 did not restore the excitation, indicating a different mechanism for ZD7288 block of toluene excitation. Note that it has been shown that ethanol activates GIRK channels (Lewohl et al., 1999), and that toluene inhibits GIRK channels (Del Re et al., 2006). It is unlikely that GABA_B_ receptors are involved, as GABA_B_ receptors are largely coupled to barium-sensitive GIRKs (Watts et al., 1996). In the presence of ZD7288, blockade of GABA receptors was sufficient to restore toluene-induced excitation, again indicating that the degree of activation of h-current is a key determinant as to whether toluene exerts a net excitatory or inhibitory effect on firing. Enhancement of GABA_A_ receptor function by toluene has been demonstrated in hippocampal slices and Xenopus oocytes expressing α1β1 GABA_A_ receptors (Beckstead et al., 2000). Additional studies will be necessary to determine whether the toluene-sensitive GABA receptors predicted by the results of the present study reside on DA VTA neurons, on VTA GABAergic interneurons, or on presynaptic inputs to DA VTA neurons.

The muscarinic antagonist atropine also significantly decreased excitation induced by 800 μM toluene, suggesting the involvement of muscarinic cholinergic receptors. In contrast, the nicotinic receptor antagonist mecamylamine did not significantly reduce toluene- or ethanol-induced excitation. Previous studies by our laboratory show that toluene inhibits native nicotinic cholinergic receptors on hippocampal neurons and recombinant forms including α4β2 expressed in Xenopus oocytes (Bale et al., 2002, 2005). Likewise, toluene inhibits muscarinic receptor-mediated calcium release from intracellular stores in neural precursor cells (Wu et al., 2002), and decreases acetylcholine release in striatum (Honma and Suda, 2004). Note that all of these effects of toluene decrease cholinergic neurotransmission, and thus would be predicted to inhibit DA VTA neuronal firing, since both muscarinic (Lacey et al., 1990) and nicotinic (Brodie, 1991; Pidoplichko et al., 1997) receptors excite these neurons. This fact, coupled with the insensitivity of toluene-induced excitation to a mixture of cholinergic and GABAergic receptor antagonists, indicates that cholinergic processes regulate toluene-induced excitation in an indirect way, possibly via effects on GABAergic transmission. Again, as with GABA receptors, determining the location of cholinergic receptors that regulate toluene-induced excitation will require extensive additional studies.

Somewhat surprisingly, we found that glutamate antagonists, even when applied in the presence of GABA and cholinergic antagonists, potentiated the excitatory effects of toluene on firing with no significant effect on ethanol excitation. While glutamate antagonists did not significantly change firing rate in the toluene experiments when applied alone, it is possible that tonic glutamate release in the slice promotes opening of glutamate-activated ion channels, resulting in a tonic reduction of membrane resistance. Via Ohm's law, blocking these receptor-generated currents would increase overall resistance, and thus boost the excitatory action of toluene. The lack of potentiation of ethanol excitation under these conditions suggests that toluene and ethanol activate DA VTA neurons through different mechanisms. These differences may be related to the relationship between glutamate receptors and the toluene-sensitive and ethanol-sensitive elements. For example, if the toluene-sensitive elements are located nearer to glutamate receptors on the neuronal membrane, there may be a more significant effect of glutamate antagonists on toluene-induced currents compared to ethanol-induced currents. Alternatively, toluene may increase glutamate release, producing a dynamic reduction in membrane resistance concurrently with the toluene-induced excitation. Additional studies will be needed to determine the mechanism by which blockade of glutamate receptors potentiates toluene-induced excitation.

Overall, the results of the studies presented here indicate that while both toluene and ethanol excite DA VTA neurons, the mechanisms underlying these effects are complex and likely reflect discrete differences in their cellular sites of action. Elucidating these diverse actions may reveal novel means by which the response of DA VTA neurons to drugs of abuse may be modulated, leading to the development of more effective pharmacotherapeutics to treat addiction.

## Author contributions

SN, CY, DA, MM, BV, and MB carried out electrophysiological recording and data analysis; SN, CY, MB, and JW participated in designing the experiments and interpretation of the results; all authors were involved in writing, reading and final approval of the submitted manuscript.

## Funding

The authors gratefully acknowledge the support of these studies by PHS Grant DA013951 and AA009986 JW and AA05846 and AA022538 MB.

### Conflict of interest statement

The authors declare that the research was conducted in the absence of any commercial or financial relationships that could be construed as a potential conflict of interest.
